# Treatment decision in adult patients with class III malocclusion: surgery versus orthodontics

**DOI:** 10.1186/s40510-018-0218-0

**Published:** 2018-08-02

**Authors:** Sara Eslami, Jorge Faber, Ali Fateh, Farnaz Sheikholaemmeh, Vincenzo Grassia, Abdolreza Jamilian

**Affiliations:** 1 Department of Orthodontics, Tehran Dental Branch, Craniomaxillofacial Research Center, Islamic Azad University, No 14, Pesiyan Ave., Vali Asr St., Tehran, 1986944768 Iran; 2 Department of Orthodontics, Faculty of Health Science, University of Brasilia, Brasilia, Brazil; 3 Craniomaxillofacial Research Center, Tehran Dental Branch, Islamic Azad University, Tehran, Iran; 4 Multidisciplinary Department of Medical-Surgical and Dental Specialties, University of Campania ‘Luigi Vanvitelli’, Naples, Italy

**Keywords:** Angle class III, Orthognathic surgery, Orthodontics

## Abstract

**Background:**

One of the most controversial issues in treatment planning of class III malocclusion patients is the choice between orthodontic camouflage and orthognathic surgery. Our aim was to delineate diagnostic measures in borderline class III cases for choosing proper treatment.

**Methods:**

The pretreatment lateral cephalograms of 65 patients exhibiting moderate skeletal class III were analyzed. The camouflage group comprised of 36 patients with the mean age of 23.5 (SD 4.8), and the surgery group comprised of 29 patients with the mean age of 24.8 years (SD 3.1). The camouflage treatment consisted of flaring of the upper incisors and retraction of the lower incisors, and the surgical group was corrected by setback of the mandible, maxillary advancement, or bimaxillary surgery. Mann-Whitney *U* test was used to compare the variables between the two groups. Stepwise discriminant analysis was applied to identify the dentoskeletal variables that best separate the groups.

**Results:**

Holdaway H angle and Wits appraisal were able to differentiate between the patients suitable for orthodontic camouflage or surgical treatment. Cases with a Holdaway angle greater than 10.3° and Wits appraisal greater than − 5.8 mm would be treated successfully by camouflage, while those with a Holdaway angle of less than 10.3° and with Wits appraisal less than − 5.8 mm can be treated surgically. Based on this model, 81.5% of our patients were properly classified.

**Conclusions:**

Holdaway H angle and Wits appraisal can be used as a critical diagnostic parameter for determining the treatment modality in class III borderline cases.

## Background

Class III malocclusion is characterized by a variety of skeletal and dental components, including a large or protrusive mandible, retrusive maxilla, protrusive mandibular dentition, retrusive maxillary dentition, and combinations of these components [1]. Its diagnosis, prognosis, and treatment have always been a challenge for clinicians [2]. A normal occlusion and improved facial esthetics of skeletal class III malocclusion can be achieved by growth modification [3], orthodontic camouflage, or orthognathic surgery [4]. The age of the patient, severity of the malocclusion, patient’s chief complaint, clinical examinations, and cephalometric analysis will delineate the treatment of choice [5]. Growth modification should begin before the pubertal growth spurt [6–10], after which only orthodontic camouflage or orthognathic surgery are possible. The severity of class III malocclusion in adult cases would define whether the patient is suitable for surgery or orthodontic treatment [11]. Kerr et al. [12] suggested that surgery should be performed in patients with ANB and incisor mandibular plane angles of lower than − 4° and 83°, respectively. Eisenhauer et al. [13] also conducted a study to separate class III patients who can be properly treated orthodontically from those who require orthognathic surgery. They suggested a predictive model including Wits appraisal, SN, maxillary/mandibular ratio, and lower gonial angle variables for correct classification of class III malocclusion in adult cases. However, problem would arise when distinguishing between borderline surgical-orthodontic class III malocclusion cases. Rabie et al. [14] evaluated borderline class III patients who had undergone camouflage orthodontic treatment or orthognathic surgery and suggested that Holdaway angle can be a reliable guide in determining the treatment modality of these patients. They further suggested that patients with a Holdaway angle greater than 12° can be successfully treated by orthodontics alone while patients with Holdaway angles less than 12° would require surgical treatment. In a similar study conducted in 2011 by Benyahia et al. [15] found a threshold or borderline value of 7.2°, thus suggesting that patients with Holdaway angles above this value can be successfully treated by orthodontics without the need for orthognathic surgery. Although both studies have shown the correlation between Holdaway angle values and the need for orthognathic surgery, the big difference between the findings of Rabie et al. [14] and Benyahia et al. [15] in estimation of the threshold value prompted us to conduct another study. Therefore, the aim of this study was to delineate diagnostic measures in borderline class III cases for choosing proper treatment modality and also to compare the treatment effects between them.

## Methods

This retrospective study was carried out in accordance with the ethical standards set forth in the 1964 Declaration of Helsinki. Informed written consent was obtained from each patient and a parent or guardian. Ethical approval with the number of 95A11181 was obtained from the Craniomaxillofacial Research Center before patient recruitment.

Lateral cephalograms of all of class III patients who had attended the private practice orthodontic office from 2011 to 2016 and met the inclusion criteria were selected for the study.

The inclusion criteria were as follows:

1. Dental class III malocclusion
2. ANB of 0° to − 4.5°; − 8.5 < Wits appraisal < − 1 mm
3. No syndromic or medically compromised patients
4. No previous surgical intervention
5. No obvious transversal discrepancy
6. No mandibular functional shift (lack of pseudo-class III)
7. Normal overjet and overbite after completion of treatment
8. Skeletally mature patients
9. Patients who have achieved adequate functional and esthetic results at the end of their treatment

By placing the significance level at 0.05 and the power at 90%, a sample size of 58 patients would be needed [16].

Out of a total number of 430 class III patients, 65 met the inclusion criteria and were selected to participate in this study. The camouflage group comprised of 36 patients (15 males and 21 females) with the mean age of 23.5 (SD 4.8) years old and confidence interval 25.6–21.2, and the surgery group comprised of 29 patients (12 males and 17 females) with the mean age of 24.8 (SD 3.1) and confidence interval 26–22.3. There was no spastically significant difference in age between groups *P* < 0.9.

Treatment of the camouflage patients included treatment with fixed orthodontic appliances in both jaws. While the majority of camouflage group patients were treated without teeth extractions, 6 of them underwent the extraction of the lower first premolars and the upper second premolars. The treatment of all of these patients was focused on flaring of the upper incisors and retraction of the lower incisors throughout class III mechanics, specially by application of class III elastics.

The patients of the surgery group also received fixed orthodontic treatment in both jaws. Nine patients had also undergone extractions of the upper first premolar and the lower second premolar teeth, while the rest were treated without extractions. Their surgical treatments were performed in the forms of either bimaxillary surgery (5 patients), maxillary advancement (16 patients), or mandibular setback (8 patients).

The pretreatment records (containing panoramic and lateral cephalograms, intra- and extra-oral photographs, and plaster models) were presented to three board-certified orthodontists. They were asked to divide the patients into the camouflage and surgery groups solely based on these records. Based on their judgment, the camouflage and surgery group consisted of 34 and 31 patients, respectively.

### Cephalometric analysis

The following cephalometric parameters were measured:

- PoOr-NBa: cranial deflexion angle
- NSAr: sella turcica angle
- BaSN: cranial base angle
- SNA: sagittal position of the maxilla relative to the anterior part of the cranial base
- SNB: sagittal position of the mandible relative to the anterior part of the cranial base
- ANB: sagittal maxillo-mandibular disparity
- Wits appraisal: sagittal disparity between Ao and Bo, orthogonal projections of A and B on the occlusal plane
- NAPog: angle showing the position of point A relative to the N-Pog facial plane
- PP-SN: inclination of the palatal plane relative to the anterior cranial base
- ML-SN: divergence of the mandibular plane relative to the anterior part of the cranial base
- Npog-SN: angle formed by the facial plane and the anterior part of the cranial base
- GoMe-SN: angle of facial divergence
- Occ/ML: inclination of the functional occlusal plane relative to the lower mandibular margin
- Occ/F: inclination of the functional occlusal plane relative to the Frankfurt plane
- PP-ML: inclination of the palatal plane relative to the lower mandibular margin
- ArGoMe: goniac angle
- Go upper or NGoAr: upper gonial angle;
- Go lower or NGoMe: lower gonial angle;
- Y-Axis: SN to S-gnathion
- U1-SN: inclination of the upper incisors relative to the anterior cranial base;
- L1-ML: inclination of the lower incisors relative to the lower mandibular margin;
- U1-L1: internal interincisal angle;
- Holdaway H angle: angle formed by soft tissue nasion–soft tissue pogonion–tangent to the upper lip
- *Z* angle: angle formed by the soft tissue pogonion–the more protrusive lip with the Frankfurt plane

All of the measurements were done separately by two skilled orthodontists. In case of any significant difference in any of the measurements, the variable would be remeasured by both of them and also a third party. The interexaminer reliability (i.e., level of agreement) between the two investigators was estimated by calculating the intraclass correlation coefficient (ICC). ICCs extended from 0.68 to 1, indicating acceptable to perfect reliability of the measurements. The magnification factor of each cephalogram was standardized at 8%.

Patient satisfaction was evaluated using the visual analog scale (VAS) [17, 18]. The subjects were asked to record their satisfaction with their facial and dental characteristics on a 10 cm VAS having phrases “very dissatisfied” (score 0) on the left end and “very satisfied” (score 10) on the right end.

### Statistical analysis

Mann-Whitney *U* test was used to compare the variables between the two groups. Stepwise discriminant analysis was applied to identify the dentoskeletal variables that best separate the groups. The discriminant function coefficients were calculated for each of the selected variables along with a constant. An equation was developed for calculating the individual scores of the patients. Discriminant analysis was also used to calculate a mean score or centroid for all patients in each group.

## Results

Mann-Whitney test showed that significant differences (*P* < 0.05) were found in eight measurements (Table 1). Stepwise discriminant analysis identified only Holdaway H angle and Wits could distinguish between patients suitable for orthodontics from those suitable for surgery. The canonical coefficient of the discriminant function and the calculated constant provided the following equation designed to calculate the individual score given to each new patient in one of the two groups:

$$ \mathrm{Group}\ \mathrm{Score}:0.232+\left(0.408\times \mathrm{Wits}\ \mathrm{appraisal}\right)\left(0.199\times \mathrm{Holdaway}\ \mathrm{H}\ \mathrm{angle}\right) $$

**Table 1:** Comparison of the pretreatment values for the between orthodontic and surgical groups

Cephalometric data	Pretreatment camouflage group		Pretreatment surgery group		Mann-Whitney test
	Mean	SD	Mean	SD	
Cranial base					
PoOr-NBa	28.5	3.6	29.7	3.3	0.394
NSAr	124.2	5.6	124.3	7.1	0.746
BaSN	128.3	4.7	130.8	6.3	0.065*
Sagittal					
SNA	79.9	3.9	79.8	3.5	0.841
SNB	81.1	4.1	82	3.4	0.352
ANB	− 1.1	1.2	− 2.1	1.2	0.001*
Wits appraisal	− 4.8	1.8	− 6.8	1.7	0.001*
NAPog	− 3.6	3.2	− 6.3	3.9	0.251
Vertical					
PP-SN	8.5	3.1	9.8	2.4	0.056*
ML-SN	35.9	13.3	36.5	4.7	0.822
Npog-SN	82.1	4.1	83.2	3.3	0.662
GoMe-SN	1.1	0.1	1.1	0.1	0.077
Occ/ML	17.6	4.1	18.2	4.4	0.588
Occ/F	8.2	3.5	7.5	3.5	0.399
PP-ML	25.5	5,5	26.6	4.8	0.383
ArGoMe	129	5.6	131.9	5.9	0.056*
Go upper or NGoAr	51.2	5.3	51.2	3.5	0.954
Go lower or NGoMe	77.4	7	80.6	4	0.01*
Y-Axis	68.6	8.6	68.1	3.8	0.797
Dental					
U1-SN	107.8	6.2	106.2	8	0.370
L1-ML	90	9.2	85.9	7.2	0.057*
U1-L1	132.4	10.3	132.8	11.2	0.872
Soft tissue					
Holdaway H angle	11.9	2.8	8.7	3.5	0.001*
*Z* angle	78	7.3	81.1	6.8	0.078

*Showed *p*<.05 was accepted as significant

The camouflage group centroid was 0.637, and the surgery group centroid was − .791. The threshold score, the mean centroid of the two groups, was − 0.077 which corresponded to Holdaway H angle of 10.3° and Wits appraisal − 5.8 (Table 2). Therefore, 81.5% of our patients were properly classified. Seven patients in the camouflage group and 5 patients in the surgical group were misclassified (Table 3).

**Table 2:** Stepwise discriminant analysis*

Predicted variables	Canonical coefficients of the discriminant function
Wits	0.408
Holdaway H angle	0.199
Constant	0.232

*Individual score: Constant + (Canonical coefficient × Holdaway H angle)

Group centroids: camouflage group 0.637, surgery group − 0.791

Threshold score − 0.077

**Table 3:** Classification results of stepwise discriminant analysis

Original group membership	Predicted group membership	
	Camouflage group	Surgery group
Camouflage group	29	7
Surgery group	5	24

No statistically significant differences were found in relation to VAS scores regarding the satisfaction of dental and facial appearance subjects (*P* < 0.855).

## Discussion

The present study investigated and focused on successfully treated borderline class III patients in order to provide some guidelines which can assist the clinicians in choosing the best treatment modality for them, namely, surgical or camouflage correction. Treatment success was assured through using cases in which the patients were satisfied with the end results. Furthermore, three board-certified orthodontists had also approved the treatment course and results of the selected cases. The severity of class III malocclusion ranges from mild dentoalveolar to severe skeletal problems. Generally, orthognathic surgery is recommended to non-growing patients with larger dentoskeletal discrepancies, while dentoalveolar compensation or camouflage is recommended for milder discrepancies; however, the decision as to which treatment should be chosen is not always an easy task specially in borderline cases. Borderline cases refer to patients with mild to moderate skeletal problems that can be treated by either orthodontic or surgical means. Also, this important fact should not be overlooked that this decision primarily belongs to the patients. Cassidy [19] defined “borderline cases” as those patients who were similar with respect to the characteristics on which the orthodontic/surgical decision appeared to have been based.

In practice, the treatment decision is based on the clinical examination and the cephalometric analysis by assessing the amount of sagittal and vertical discrepancy, dentoalveolar compensations, and facial esthetics. The results of this study confirmed the importance of facial esthetics in the class III decision-making process. The Holdaway H angle was singled out by discriminant analysis as being the decisive parameter. The threshold or borderline value for Holdaway and Wits appraisal were 10.3° and − 5.8 mm, respectively. In 1983, Holdaway [20] defined this angle as being formed by the soft tissue H line and the soft tissue facial plane (Na-Pog). Ideally, its value is 10° when facial convexity is normal. This angle quantifies the protrusion of the upper lip relative to soft tissue profile and is independent of the skeletal discrepancy of the bases (ANB angle). Consequently, it is perfect for characterizing the profile of borderline surgical skeletal class III, in whom esthetics and facial appearance might be of greater importance than occlusion or skeletal discrepancy.

Therefore, the finding of this study implies that a new borderline class III malocclusion patient with a Holdaway angle greater than 10.3° would be treated successfully by camouflage alone, while a new patient with a Holdaway angle of less than 10.3° should be treated by combined surgery. This study also showed that Wits appraisal greater than − 5.8 mm would be effectively corrected by camouflage and less than − 5.8 mm must be treated by surgery. In this way, 81.5% of our patients were properly classified. On the contrary, Rabie et al. [14] suggested that patients with a Holdaway angle greater than 12° can be successfully treated by orthodontics alone while patients with Holdaway angles less than 12° would require surgical treatment. In a similar study, Benyahia et al. [15] reported this critical angle as 7.2°. The differences between these results could be due to different inclusion criteria. Selection bias with recruitment was avoided by including consecutive cases from database of completed cases of a clinic. Moreover, this study was a retrospective one, and all the samples met the inclusion criteria. All the patients were treated by one orthodontist, and one surgeon operated on them.

The treatment of all patients in camouflage group was focused on flaring of the upper incisors and retraction of the lower incisors throughout class III mechanics, specially by application of class III elastics. No bone-anchored appliance was used in this group. One of the weaknesses of this study is the variety in the surgical procedures. Further research is needed with no variety in the surgical procedures.

Kerr et al. [12] tried to establish cephalometric yardsticks to objectify the decision for treatment. The important factors that differentiated the surgery and orthodontic patients in their study were the size of the antero-posterior discrepancy, the inclination of the mandibular incisors, and the appearance of the soft tissue profile. Also, Ghiz [21] presented a logistic equation with four variables to predict the future success of early orthopedic treatment and could correctly classify 95.5% of the successfully treated infants but only 70% of the unsuccessfully treated infants.

In a similar study, Eisenhauer showed that the Wits appraisal is the most decisive parameter for determining orthodontic therapy or orthognathic surgery in adult patients with class III malocclusion [13]. Recently, Martinez reported that Wits appraisal, lower incisor inclination, and inter-incisal angle were indicative in treatment of camouflage or orthognathic surgery [22].

## Conclusions

This study found that borderline class III malocclusion patients who have a Holdaway angle greater than 10.3° would be treated successfully by camouflage alone, while surgery should be the treatment of choice in borderline class III malocclusion patients with a Holdaway angle of less than 10.3°. This study also showed that Wits appraisal greater than − 5.8 mm would be effectively corrected by camouflage and less than − 5.8 mm must be treated by surgery.
